# Structure and Distribution of an Unrecognized Interstitium in Human Tissues

**DOI:** 10.1038/s41598-018-23062-6

**Published:** 2018-03-27

**Authors:** Petros C. Benias, Rebecca G. Wells, Bridget Sackey-Aboagye, Heather Klavan, Jason Reidy, Darren Buonocore, Markus Miranda, Susan Kornacki, Michael Wayne, David L. Carr-Locke, Neil D. Theise

**Affiliations:** 1 Department of Medicine, Division of Digestive Diseases, Mount Sinai Beth Israel Medical Center, Icahn School of Medicine at Mount Sinai, New York, New York, 10003 USA; 2 Department of Medicine, Division of Gastroenterology, Zucker School of Medicine at Hofstra/Northwell, 500 Hofstra Blvd, Hempstead, NY 11549 USA; 3 Department of Medicine, Division of Gastroenterology, Perelman School of Medicine, University of Pennsylvania, Philadelphia, Pennsylvania 19104 USA; 4 Department of Bioengineering and Center for Engineering MechanoBiology, School of Engineering and Applied Sciences, University of Pennsylvania, Philadelphia, Pennsylvania 19104 USA; 5 Department of Pathology, Mount Sinai Beth Israel Medical Center, Icahn School of Medicine at Mount Sinai, New York, New York, 10003 USA; 6 Department of Pathology, New York University School of Medicine, New York, New York, 10016 USA; 7 Department of Surgery, Mount Sinai Beth Israel Medical Center, Icahn School of Medicine at Mount Sinai, New York, New York, 10003 USA; 8 The Center for Advanced Digestive Care, Weill Cornell Medicine, New York Presbyterian Hospital, 1305 York Avenue, 4th Floor, New York, New York, 10021 USA

## Abstract

Confocal laser endomicroscopy (pCLE) provides real-time histologic imaging of human tissues at a depth of 60–70 μm during endoscopy. pCLE of the extrahepatic bile duct after fluorescein injection demonstrated a reticular pattern within fluorescein-filled sinuses that had no known anatomical correlate. Freezing biopsy tissue before fixation preserved the anatomy of this structure, demonstrating that it is part of the submucosa and a previously unappreciated fluid-filled interstitial space, draining to lymph nodes and supported by a complex network of thick collagen bundles. These bundles are intermittently lined on one side by fibroblast-like cells that stain with endothelial markers and vimentin, although there is a highly unusual and extensive unlined interface between the matrix proteins of the bundles and the surrounding fluid. We observed similar structures in numerous tissues that are subject to intermittent or rhythmic compression, including the submucosae of the entire gastrointestinal tract and urinary bladder, the dermis, the peri-bronchial and peri-arterial soft tissues, and fascia. These anatomic structures may be important in cancer metastasis, edema, fibrosis, and mechanical functioning of many or all tissues and organs. In sum, we describe the anatomy and histology of a previously unrecognized, though widespread, macroscopic, fluid-filled space within and between tissues, a novel expansion and specification of the concept of the human interstitium.

## Introduction

The interstitial space is the primary source of lymph and is a major fluid compartment in the body. While the anatomy and composition of the interstitial space between cells is increasingly understood, the existence, location, and structure of larger inter- and intra-tissue spaces is described only vaguely in the literature. This is particularly important in reference to “third spacing” (interstitial fluid build-up) and when considering overall interstitial fluid flow and volume, which have not been well studied^1^.

**Figure 1:**
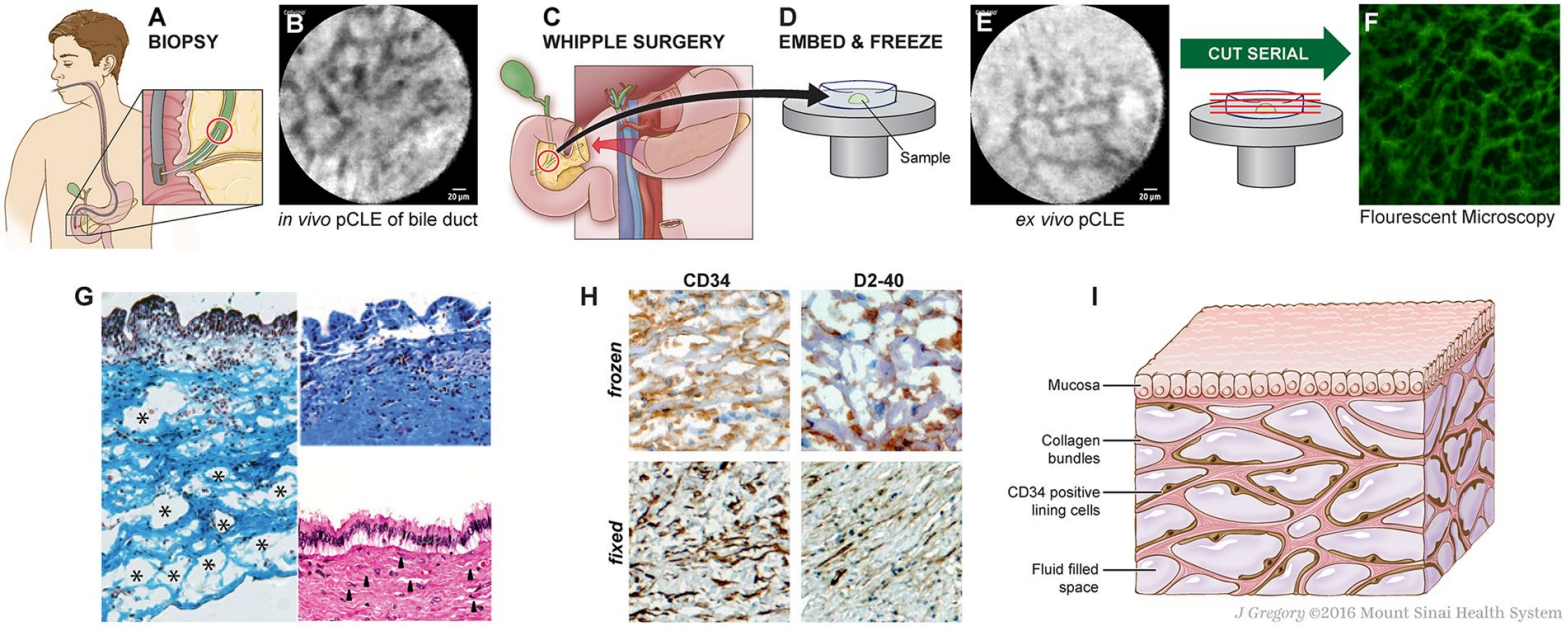
Identification of bile duct reticular pattern and demonstration of submucosal space. (**A**,**B**) pCLE of bile duct after fluorescein injection shows a reticular pattern at a depth of 60–70 μm. Scale bar, 20 μm. (**C–E**) Bile duct tissue removed at the time of Whipple surgery was frozen and *ex vivo* pCLE performed, demonstrating persistence of the reticular pattern. Scale bar, 20 μm. (**F**) Unstained frozen tissue of submucosa of a bile duct imaged by fluorescent microscopy, showing the reticular pattern in this layer of bile duct wall. The “bright” spaces are now dark (fluoresceinated fluid drained in processing and the tissue structures remained stained with residual fluorescein). (**G**) Masson trichrome of fresh-frozen bile duct shows that the dark bands are collagen bundles (blue) (left). The upper right shows Masson trichrome of a normally processed/fixed bile duct from the same patient, with collapse of spaces and apparent adherence of collagen bundles to each other. Lower right shows the fixed specimen stained with H&E; the thin spaces between collagen layers (arrows) reflect normally fluid-filled spaces that are almost completely collapsed. (**H**) Frozen (top) and fixed (bottom) bile ducts immunostained with antibodies against CD34 (left, brown) and D2-40 (right, brown) show cells lining the collagen bundles; note that bundles often seem to have a lining cell on one side, but not the other (20×, DAB, hematoxylin). (**I**) Schematic of the fluid-filled space supported by a network of collagen bundles lined on one side with cells. *Illustration by Jill Gregory*. *Printed with permission from Mount Sinai Health System*, *licenced under CC-BY-ND*. (https://creativecommons.org/licenses/by-nd/4.0/legalcode).

Advances in *in vivo* microscopy offer the potential to identify new, functionally-relevant anatomical structures in humans. Lymphatic vessels in the brain, for example, were recently identified for the first time using *in vivo* multiphoton microscopy imaging through a thinned skull preparation^2^. Probe-based Confocal Laser Endomicroscopy (pCLE) is an *in vivo* imaging technology that provides real-time histologic assessment of tissue structures during endoscopy, generally after intravenous injection of fluorescein. We and others have observed that, in the extrahepatic bile ducts and pancreatic ducts, pCLE at the fixed focal length of 60–70 μm shows a submucosal “reticular pattern” (Fig. 1A,B) consisting of 20 μm wide dark branching bands surrounding large, fluorescein-filled polygonal spaces^3^. These have no obvious correlate to known structures. Although endoscopists have suggested that this network represents capillaries or lymphangioles^3^, neither structure can explain the reticular pattern of dark bands and bright, fluid-filled spaces.

We hypothesized that this pattern reflects an extension of the intercellular interstitial space. We carried out an in-depth study using pCLE and histology of the human extrahepatic bile duct in order to identify the microanatomic correlates of the reticular pattern. We report here the existence of a novel interstitial (*i*.*e*. pre-lymphatic) space defined by a complex lattice of thick collagen bundles. We observed similar structures when we extended our study to include the dermis, peri-arterial stroma, submucosa of the viscera (gastrointestinal tract, urinary bladder), bronchial tree of the lungs, and fascial planes of the musculoskeletal system and adipose tissue, and as a result propose a large-scale revision of the macro- and microanatomy of the human interstitium.

## Results

Samples were obtained from surgical specimens of bile ducts resected during twelve pancreatico-biliary surgeries. Several minutes prior to vascular ligation and resection of the surgical specimen, patients were infused intravascularly with fluorescein with direct *in situ* visualization by pCLE of the reticular pattern (Fig. 1A,B). The site was resected and then immediately scanned again with pCLE *ex vivo*, confirming that the reticular pattern and the fluorescein were still intact after resection (data not shown). The specimens were then embedded in frozen section medium, rapidly frozen using an aerosol-based freezing spray, and re-imaged (Fig. 1C,D,E). Serial frozen sections were cut perpendicular to the viewing angle of the pCLE exactly *en face* to the lumen surface, marking sections every 5 μm until a depth of 60–70 μm was reached. We also performed serial sections of tissue in a cross-sectional plane. These sections were visualized under routine fluorescence microscopy (Fig. 1F), showing that the reticular pattern correlated with thick, fluorescein-stained bundles (seen on pCLE as black bands). The absence of fluorescence between these bundles in the final slides is due to the process of frozen section slide preparation (air drying, fixation and washing) causing the fluid to drain from the final slide.

Parallel frozen sections were stained with Masson’s trichrome stain, confirming the presence of collagenous bands separating open, formerly fluid-filled spaces (Fig. 1G, left). These structures co-localized with the compacted biliary submucosa normally seen in biopsy and resection specimens (Fig. 1G, right, from the same patient as sample on left). This suggests that the previously described dense structure of the submucosa represents an artifact due to loss of fluid during tissue excision and fixation, causing normally-separated collagen bundles to collapse and adhere to each other. We observed that the reticular pattern appeared within 30 seconds of intravascular infusion of fluorescein, approximately the same time point at which lymph nodes are visualized, but later than when vascular structures are visualized^4^. This suggests that it is a form of interstitial space in which interstitial fluid or “pre-lymph” accumulates or forms. Immunostaining of the frozen and fixed bile duct submucosa showed positive CD34 and D2-40 staining on one side of each collagen bundle (Fig. 1H). Immunostaining was negative for other lymphovascular endothelial markers (CD31, ERG, LYVE-1), but uniformly positive for the mesenchymal marker vimentin (data not shown). Stains for the myoepithelial marker smooth muscle actin, the stem cell marker CD117 and nuclear beta-catenin were negative (data not shown). Figure 1I is a schematic summarizing the histological observations.

**Figure 2:**
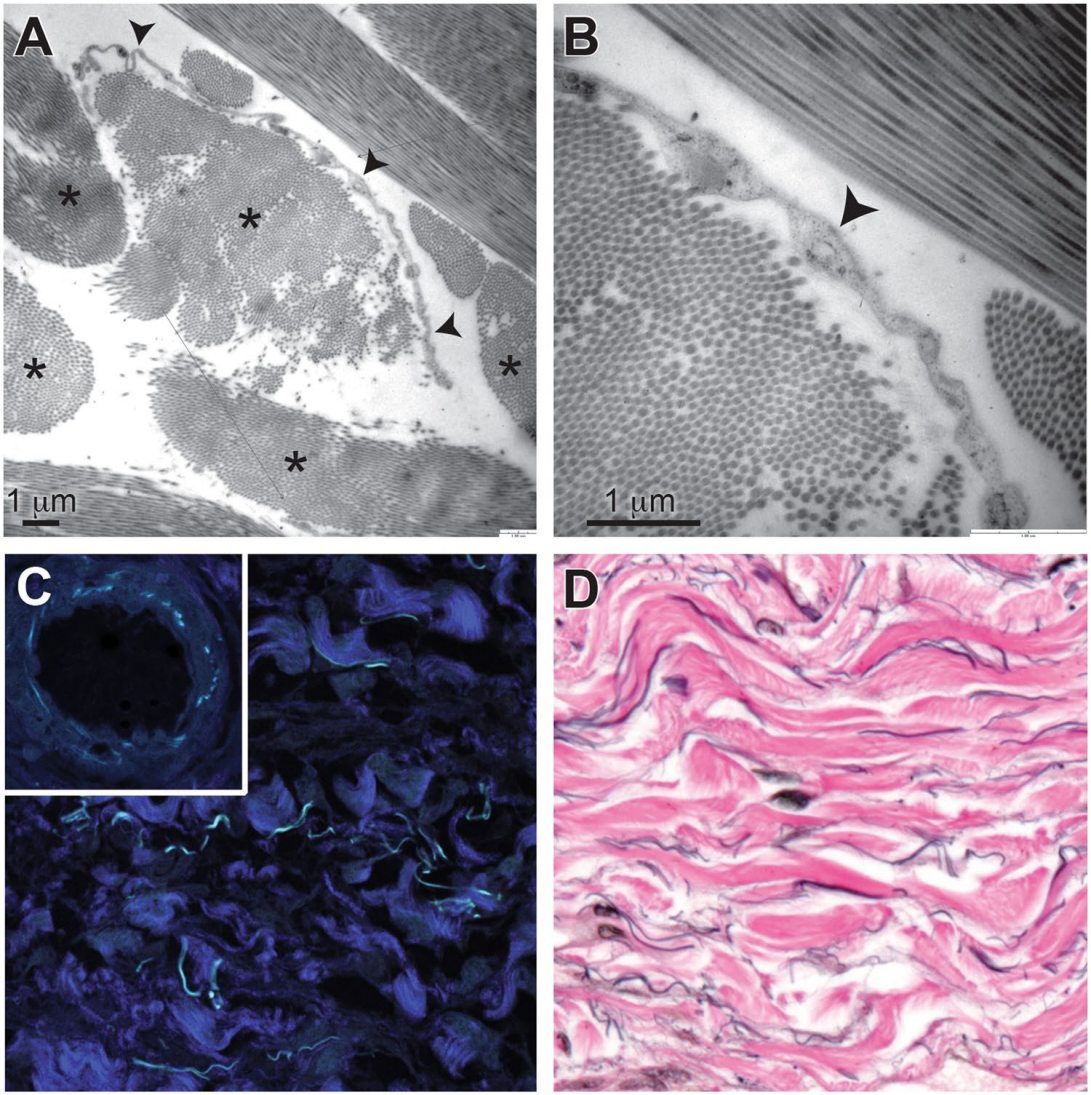
Structural evaluation of the interstitial space. (**A**) Transmission electron microscopy shows collagen bundles (asterisks) that are composed of well-organized collagen fibrils. Some collagen bundles have a single flat cell along one side (arrowheads). Scale bar, 1 μm. (**B**) Higher magnification shows that cells (arrowhead) lack features of endothelium or other types of cells and have no basement membrane. Scale bar, 1 μm. (**C**) Second harmonics generation imaging shows that the bundles are fibrillar collagen (dark blue). Cyan-colored fibers are from autofluorescence and are likely elastin, as shown by similar autofluorescence in the elastic lamina of a nearby artery (inset) (40×). (**D**) Elastic van Gieson stain shows elastin fibers (black) running along collagen bundles (pink) (40×).

Ultrastructural studies (Fig. 2A,B) show that the collagen bundles are asymmetrically lined on one side by thin, flat cells (spindle shaped in cross section) that have scant cytoplasm and an oblong nucleus. These cells are fibroblast-like, without cell-type specific structures; in particular, they are devoid of ultrastructural features indicative of endothelial differentiation, including pinocytotic vesicles and Weibel-Palade bodies^5^. Electron microscopy also shows that these cells have no basement membrane – suggesting that they adhere directly to the underlying collagen bundles – and that, while one side of a given collagen bundle is lined by these cells, the opposite side is usually unlined and directly exposed to fluid in the space. The bundles are well-visualized by second harmonics generation imaging (Fig. 2C), confirming that they are fibrillar collagen^6^. Autofluorescent elastic fibers are also observed, as confirmed by the similar appearance of the elastic lamina in arteries in the same tissue (Fig. 2C inset) and by an elastic Van Gieson histochemical stain (Fig. 2D).

**Figure 3:**
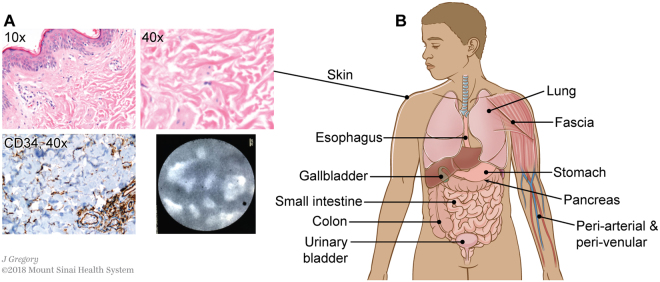
An interstitial space is found in the dermis and submucosae and other fibroconnective tissues throughout the body. (**A**) Skin stained with H&E (upper left 10×, upper right 40×) shows the same structures as identified in the extrahepatic bile duct. Immunostain for CD34 (lower left, brown DAB, light blue hematoxylin counterstain, 40×) highlights that the lining cells are intermittent and often on one side of the collagen bundles, but not the other. pCLE applied to the skin *in vivo* following this histologic observation confirms that the histologic appearance predicts the *in vivo* reticular pattern when pCLE is applied to the skin. (**B**) Schematic showing location of identical histologic structures seen in fibroconnective tissues throughout the body (see Supplemental Fig. 1 for histology images). *Illustration by Jill Gregory*. *Printed with permission from Mount Sinai Health System*, *licenced under CC-BY-ND*. (https://creativecommons.org/licenses/by-nd/4.0/legalcode).

The characteristic histological features of this bile duct submucosa structure (spaces filled with fluid and with collagen bundles lined asymmetrically by flat cells) are readily visualized in other tissues. The structure was recognized consistently in the dermis in clinical resection specimens of skin (Fig. 3A), and pCLE applied to thin regions of skin *in vivo* after fluorescein injection showed the same reticular pattern in the dermis as in the bile duct. Fluid-filled spaces and collagen bundles lined by cells staining for CD34 are seen on histology in multiple organs and tissues, including in the submucosa of the entire digestive tract, the urinary bladder, peribronchial tissue, fascia, and stroma of arteries and veins of all sizes (Fig. 3B and Supplemental Fig. 1).

**Figure 4:**
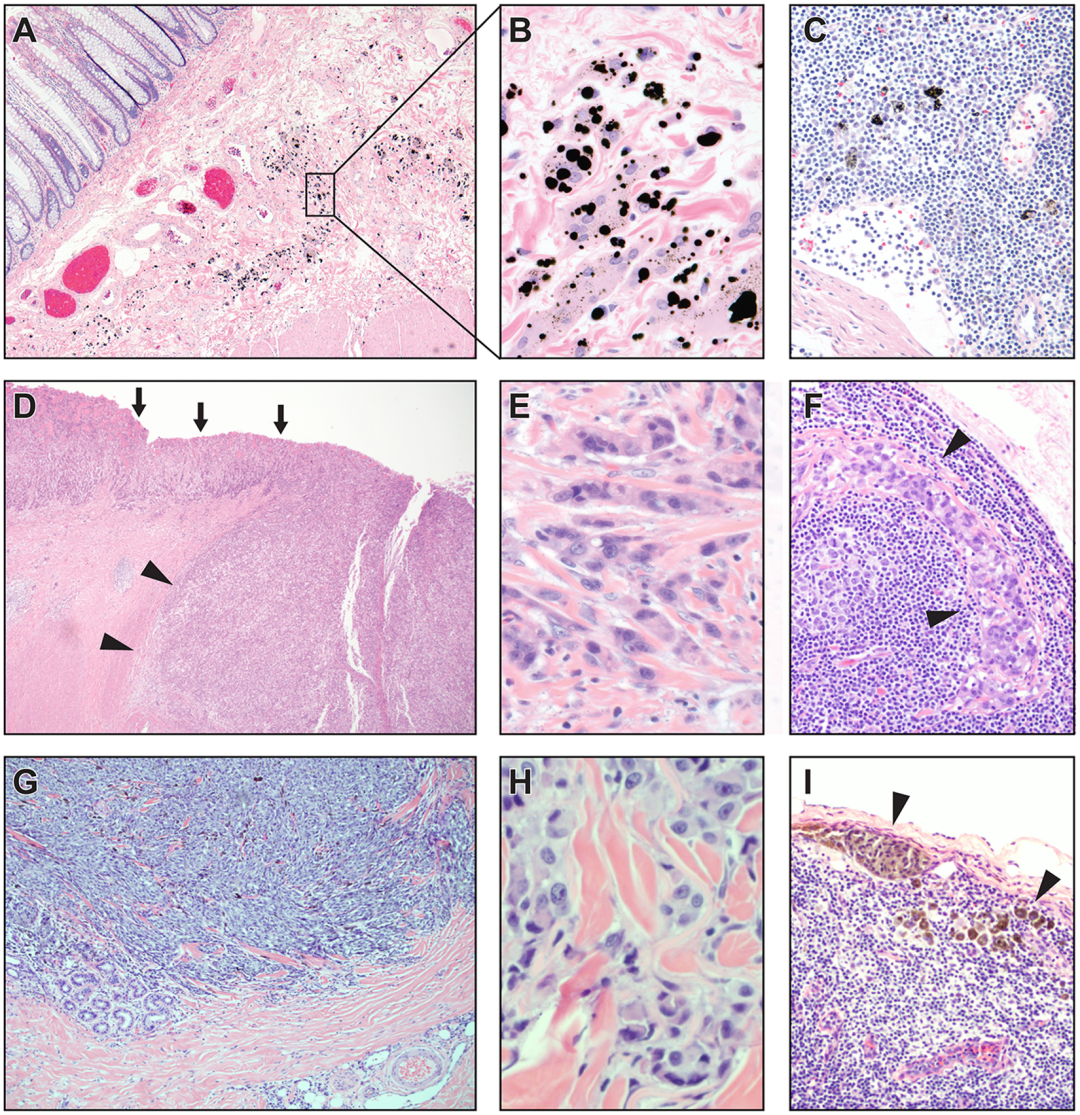
Continuity between interstitium and draining lymphatics. (**A–C**) Colon tissue with submucosal tattoo. (**A**) Black pigment endoscopically injected into submucosa of colonic wall before resection of colonic malignancy (H&E, 10×). (**B**) Black pigment is present in macrophages in the spaces between collagen bundles (H&E, 40×). (**C**) Pigment-containing macrophages are present in mesenteric lymph nodes draining the tattooed colon, showing that the interstitial space functionally communicates with lymphatic drainage of the colon (H&E, 20×). Typical images from 4 independent samples evaluated. (**D–F**) Stage T2 gastric carcinoma, poorly differentiated. (**D**) Gastric carcinoma present at the mucosal surface (arrows) invades into the submucosa (arrow heads); deeper invasion and lymphovascular invasion were not seen (H&E, 4×). (**E**) Poorly differentiated tumor cells infiltrate, singly and in very small clusters, through the interstitial space of the gastric submucosa, isolating pre-existing collagen bundles (H&E, 40×). (**F**) Metastatic carcinoma in draining mesenteric lymph nodes of the gastric resection specimen; no other metastases were identified clinically or histologically (H&E, 20×). (**G–I**) Stage T2 malignant melanoma of the skin of the left arm. (**G**) Malignant melanoma (dark blue) invading into the dermis; lymphovascular invasion not identified (H&E, 4×). (**H**) Malignant melanoma cells infiltrate, singly and in very small clusters, through the interstitial space of the dermis, isolating pre-existing collagen bundles (H&E, 40×). (**I**) Metastatic malignant melanoma in draining axillary lymph nodes; no other metastases were identified clinically or histologically (H&E, 10×).

These structures appear to be pre-lymphatic spaces, as suggested by examining colorectal cancer resection specimens with submucosal tattoos (Fig. 4A) in which black pigment is present in macrophages within the interstitial space (Fig. 4B) as well as in macrophages in associated, draining mesenteric lymph nodes (Fig. 4C). These macrophages are not seen in interstitial spaces in examination of normal tissues and are presumed to traffic into the interstitium in response to the presence of foreign material. Additionally, examination of small intestinal resection specimens from incarcerated hernias with obstructed proximal bowel shows a submucosa with diffuse splaying of the collagen bundles by proteinaceous (pink) fluid (Supplemental Fig. 2) histologically similar to lymph.

The pre-lymphatic nature of the space is further emphasized by study of stage T2 invasive tumors of the stomach (Fig. 4D–F, n = 3) and skin (Fig. 4G–I, n = 2). Stage T2 invasion of the stomach is defined as invasion into the submucosa, but no deeper. T2 invasion into the skin, likewise, indicates invasion directly into the dermis, but no deeper. Invasion by poorly differentiated invasive gastric carcinoma (Fig. 4D) shows spread through the interstitial space, surrounding still intact collagen bundles (Fig. 4E); despite no demonstrable lymphovascular invasion in this specimen, there is metastasis to a draining mesenteric lymph node (Fig. 4F). In a malignant melanoma arising on the upper arm and invading directly into dermis (Fig. 4G), there is similar spread through the interstitial space with isolation of collagen bundles (Fig. 4H); again, despite absence of demonstrable lymphovascular invasion, there is metastasis to a draining axillary lymph node (Fig. 4I). In both cases, there were no other lesions or routes of spread identified even after complete clinical and histologic examination of the patients.

## Discussion

We propose here a revision of the anatomical concepts of the submucosa, dermis, fascia, and vascular adventitia, suggesting that, rather than being densely-packed barrier-like walls of collagen, they are fluid-filled interstitial spaces. The presence of fluid has important implications for tissue function and pathology. Our data comparing rapidly-biopsied and frozen tissue with tissue fixed in a standard fashion suggest that the spaces we describe, supported and organized by a collagen lattice, are compressible and distensible and may thus serve as shock absorbers. All of the organs in which we have detected this structure are subject to cycles of compression and distension, whether relatively constant (lungs, aorta) or intermittent (digestive tract after a meal, urinary bladder during micturition, skin under mechanical compression, fascial planes during action of the musculoskeletal system). The dermal interstitium and the fascial interstitium may be mechanistically important in explaining edema (as with the pre-obstructed bowel in Supplemental Fig. 2). “Third spacing” in post-operative lymphedema (as when draining nodes are excised) and anasarca due to liver, renal, or cardiac failure may reflect fluid distention and stasis in this interstitial space. The submucosal interstitial space of the biliary tree – extending through the full extent of the extra- and intra-hepatic portal stroma – may explain the characteristic duct edema that rapidly develops in acute large bile duct obstruction.

Further support for the relationship between the histology we observe and *in vivo* structure comes from ultrasonography of tissues. Endoscopic ultrasound of the bile duct shows that it consists of three layers, the middle one of which comprises 90% of the wall thickness and is fluid filled^7^; it corresponds to the submucosal interstitium. Submucosal spaces of other viscera, the dermis, and fascia appear heterogeneous by ultrasound, typically indicative of fluid or adipose tissue, while truly dense collagenized stroma, such as in tendons and ligaments, appears dark by ultrasound^8–11^. Additional support for our observations is found in published ultrastructural studies of skin, vermiform appendix, and peri-aortic adventitia, which also appear to have included these structures, although they were not well characterized^12–14^. In the liver, the previously identified “space of Mall” in the portal region may represent this interstitium^15^. Indeed, Mall’s original drawings, derived from injection studies, appear to represent the same structures we identify here^16^.

The nature of the lining cells is unclear. While cells of the extrahepatic bile duct stained for both CD34 and D2-40, D2-40 staining was absent in all other tissues examined. Vascular endothelial cells also co-express CD34 and vimentin, but the lack of endothelial features on electron microscopy excludes this classification for the interstitial lining cells we observe, suggesting instead that they are a novel, CD34-positive form of fibroblast or even mesenchymal stem cell^17^. Whether they are the cells that deposit the collagen bundles is unknown; if so, they would be important in scar formation in wound healing. Notably, keloid scars show collagen bundles and large spaces that appear to be an exaggeration of these structures in the underlying dermis^18^. Recent data showing that keloid scars appear in regions of skin under high tension raise questions about the impact of mechanical forces and fluid flow on the structures and cells of this space^19^.

It is likely that the submucosal interstitium we describe corresponds to the interstitial spaces described in studies of metastasizing cell clusters^20^. The presence of a network of submucosal channels in the digestive and urinary tracts could explain the greatly increased likelihood of metastasis by luminal invasive tumors once they reach the submucosa. As illustrated by the cases we show of invasive melanoma and gastric cancer (Fig. 4D–I), the presence of submucosal/dermal fluid-filled channels also suggests the reason for T2 lesions being at such significantly increased risk for metastasis over stage T1 lesions – because visceral submucosae and the dermis are open, fluid filled spaces, rather than a wall of dense of connective tissue, they may be easily traveled by invasive tumor cells. Moreover, the mechanical pressure on such spaces (peristalsis in the digestive tract, compression and/or movement-associated pressure on skin) could further promote spread through these spaces. If the interstitial lining cells are the precursors of fibrogenic myofibroblasts, they might also function as first responders in peri-tumoral sclerosis of the pancreatico-biliary tree, tubular digestive tract, bronchial tree, urinary bladder and skin. Indeed, a unique population of CD34/vimentin co-expressing peritumoral fibroblasts has been reported^21^. These cells could also serve important roles in non-malignant sclerotic conditions including biliary atresia and primary sclerosing cholangitis in the biliary tree, scleroderma in the dermis and esophagus, and inflammatory bowel disease in the digestive tract. Ongoing studies are focused on characterizing these cells and their functions.

The flow of interstitial fluid through the submucosal space of the luminal GI tract is likely guided by peristalsis, in parallel with luminal contents. If there is communication between the gut lumen and the submucosal space, this raises the possibility that cell signaling (including hormonal or immunologic signals) could be regulated in a proximal-to-distal manner determined by the speed of peristalsis. Immunologic interactions in this interstitial space could also be important in inflammatory conditions such as primary sclerosing cholangitis, chronic pancreatitis, inflammatory bowel disease and scleroderma. Interestingly, while macrophages were not seen in these spaces in the normal tissues examined, they clearly traffic into the space to take up the tattoo pigment after submucosal injection (Fig. 4A–C).

The collagen bundles in the interstitial space are lined on only one side by cells, implying that the collagen matrix on the opposite side is in direct contact with interstitial fluid. There are few examples known in the human body other than the interstitial space between cells, the renal glomerulus and the space of Disse, where fluid is in direct contact with matrix proteins without an intervening cell barrier. Collagen fibers, which are charged molecules, may form an important physiologically active surface. Whether cells of the immune system or other cells passing through the space interact with the collagen bundles is a highly physiologically relevant question that requires further investigation.

In sum, while typical descriptions of the interstitium suggest spaces between cells, we describe macroscopically visible spaces within tissues – dynamically compressible and distensible sinuses through which interstitial fluid flows around the body. Our findings necessitate reconsideration of many of the normal functional activities of different organs and of disordered fluid dynamics in the setting of disease, including fibrosis and metastasis. A submucosa subjected to directional, peristaltic flow is not the previously envisaged wall of dense connective tissue, but a potential conduit for movement of injurious agents, pro-fibrogenic signaling molecules, and tumor cells. This raises the possibility that direct sampling of the interstitial fluid could be a diagnostic tool. Finally, our study demonstrates the power of *in vivo* microscopy to generate fresh insights into the anatomy and physiology of normal and diseased tissues.

## Methods

### Patients and tissue specimens

Thirteen patients undergoing surgical resection of the biliary tree at Mount Sinai Beth Israel were recruited to participate in this study between July 2012 and Dec 2013. The study was conducted with the approval of and in accordance with the relevant guidelines and regulations of the Icahn School of Medicine at Mount Sinai (Mount Sinai Beth Israel Medical Center) Institutional Review Board (IRB). Written informed consent was obtained from all patients for participation in the study (including intraoperative injection with fluorescein and pCLE) and examination of the resected specimen under microscopy to assess surgical margins with probe based confocal laser endomicroscopy (pCLE). Inclusion criteria included the ability to give informed consent and age above 18 years old. Additional tissues for study from other organs were obtained from fixed archival materials, exempt from IRB review.

### Intraoperative and Perioperative Assessment

Patients were infused with 2.5 mL of 10% fluorescein just prior to vascular ligation and specimen removal (which took approximately 5–10 minutes in all cases). Peripheral bile duct, pancreatic duct and duodenal wall samples of the final resection specimens were then studied further.

Samples were obtained from patients who were injected with fluorescein (common bile duct = 10, pancreatic duct = 3, duodenum = 3, lymph node = 1) and from uninjected control patients (common bile duct = 3, pancreatic duct = 1, duodenum = 1, lymph node = 1). Some surgical samples were large enough to enable us to study several different tissues.

### Sample Processing and Evaluation

Specimens were scanned with pCLE (Mauna Kea Technologies, Cellvizio® Cholangioflex miniprobe) *ex vivo* immediately after removal, confirming that the reticular pattern and the fluorescein were intact. Specimens were then embedded in glycerin-based OCT freezing medium and rapidly frozen using an aerosol-based freezing spray (Thomas® Cyto-Freeze) to ensure fluorescein retention and minimal water crystal formation. The specimens were either embedded with the luminal side up or in cross section. Some specimens were large enough to be bisected and evaluated in both planes. All specimens were stored at −20 °C. Beginning at the luminal surface of the *en face* bile ducts and pancreatic ducts, frozen sections were cut consecutively at 5 μm thickness to a depth of 60–70 μm. We also performed serial sections of tissue in a cross-sectional plane to the bile duct.

Specimens were mounted on glass slides without fixative and air-dried, after which they were treated using a xylene-based frozen section protocol, coverslipped and evaluated and photographed using a fluorescent microscope with a FITC excitation filter (475–490 nanometers).

### Immunohistochemical staining

Immunostaining was performed according to standard procedures^22^. Fixed specimens were first deparaffinized. Frozen section specimens were air-dried and heat fixed. After overnight incubation at 4 °C with primary antibody (supplemental Table 1) and washing with PBS, 4 μm sections were subjected to biotinylated secondary antibody and thereafter to avidin-biotinylated enzyme reagent (avidin-biotinylated horse radish peroxidase). After incubation with 1–3 drops peroxidase substrate (DAB-3,30-diaminobenzidin; 0.6 mg/ml H2O20,03% in 0.01 M Tris-HCl buffer pH 7.6) and subsequent washing in deionized H_2_O, sections were counterstained with Gill’s hematoxylin and mounted under xylene-based crystal mount.

### Electron Microscopy

Specimens for Electron Microscopy were fixed in Karnovsky fixative, postfixed in 1% phosphate-buffered osmium tetroxide, and embedded in standard fashion. ~80 nm epoxy sections were cut on an AO/Reichert Ultracut, stained with uranyl and lead salts, and examined in a Zeiss EM 900 electron microscope @80 kV.

### Second Harmonic Generation Analysis

Collagen second harmonic generation (SHG) microscopy was performed on unstained slides using a Leica TCS-SP5 Confocal/Multiphoton Microscope with a Coherent Chameleon Ultra II laser tuned to a wavelength of 900 nm. Images were acquired with non-descanned detectors.

### Data Availability

No datasets were generated or analyzed during the current study.

## Electronic supplementary material

Supplementary Figures and Legends 1 and 2
