# Author Correction: Structure and Distribution of an Unrecognized Interstitium in Human Tissues

**DOI:** 10.1038/s41598-018-25732-x

**Published:** 2018-05-10

**Authors:** Petros C. Benias, Rebecca G. Wells, Bridget Sackey-Aboagye, Heather Klavan, Jason Reidy, Darren Buonocore, Markus Miranda, Susan Kornacki, Michael Wayne, David L. Carr-Locke, Neil D. Theise

**Affiliations:** 1 Department of Medicine, Division of Digestive Diseases, Mount Sinai Beth Israel Medical Center, Icahn School of Medicine at Mount Sinai, New York, New York 10003 USA; 2 Department of Medicine, Division of Gastroenterology, Zucker School of Medicine at Hofstra/Northwell, 500 Hofstra Blvd, Hempstead, NY 11549 USA; 3 Department of Medicine, Division of Gastroenterology, Perelman School of Medicine, University of Pennsylvania, Philadelphia, Pennsylvania 19104 USA; 4 Department of Bioengineering and Center for Engineering MechanoBiology, School of Engineering and Applied Sciences, University of Pennsylvania, Philadelphia, Pennsylvania 19104 USA; 5 Department of Pathology, Mount Sinai Beth Israel Medical Center, Icahn School of Medicine at Mount Sinai, New York, New York 10003 USA; 6 Department of Pathology, New York University School of Medicine, New York, New York 10016 USA; 7 Department of Surgery, Mount Sinai Beth Israel Medical Center, Icahn School of Medicine at Mount Sinai, New York, New York 10003 USA; 8 The Center for Advanced Digestive Care, Weill Cornell Medicine, New York Presbyterian Hospital, 1305 York Avenue, 4th Floor, New York, New York 10021 USA

Correction to: *Scientific Reports* 10.1038/s41598-018-23062-6, published online 27 March 2018

The Supplementary Figure file that accompanies this Article contains an error in Supplementary Figure S1, where the Small Intestine CD34 panel was duplicated from the Gallbladder CD34 panel. The correct Figure S1 appears below as Figure 1.

**Figure 1:**
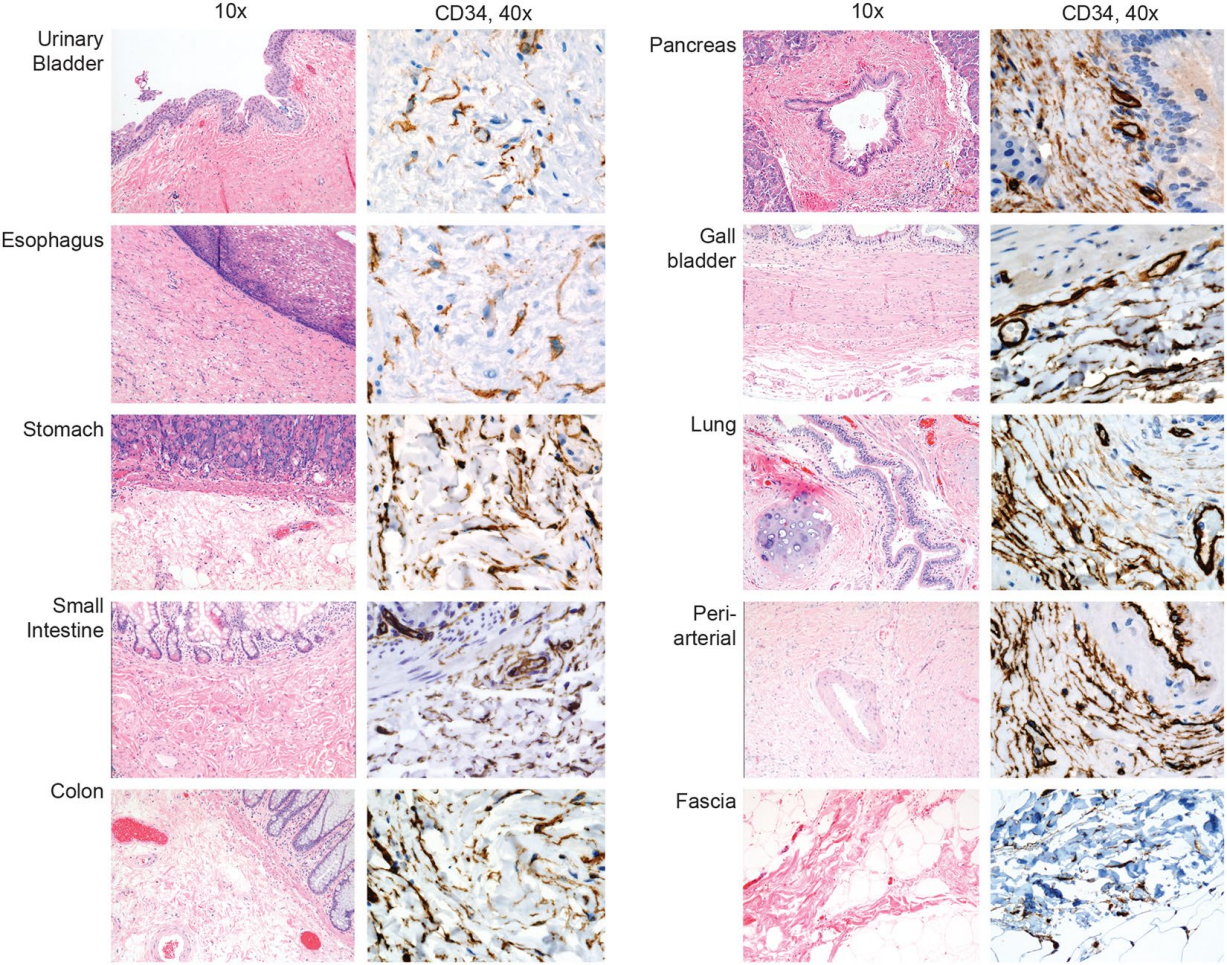
.

