# Supplementary material for: Structure and Distribution of an Unrecognized Interstitium in Human Tissues
Source: Sci Rep. 2018 Mar 27;8:4947. doi: 10.1038/s41598-018-23062-6 (PMC5869738; doi:10.1038/s41598-018-23062-6)
Supplement: Supplementary file 1 — Supplementary Figures and Legends 1 and 2 [file 41598_2018_23062_MOESM1_ESM.pdf]

STRUCTURE AND DISTRIBUTION OF AN UNRECOGNIZED INTERSTITIUM IN HUMAN TISSUES

Petros C. Benias, MD*[a], Rebecca G. Wells, MD*[b,c], Bridget Sackey-Aboagye, PhD[b], Heather Klavan, MD[a], Jason Reidy, PhD[d], Darren Buonocore, MD[d],

Markus Miranda[a], Susan Kornacki[e] Michael Wayne, DO[f],

David L. Carr-Locke, MD[a], Neil D. Theise, MD**[a,d,e]**

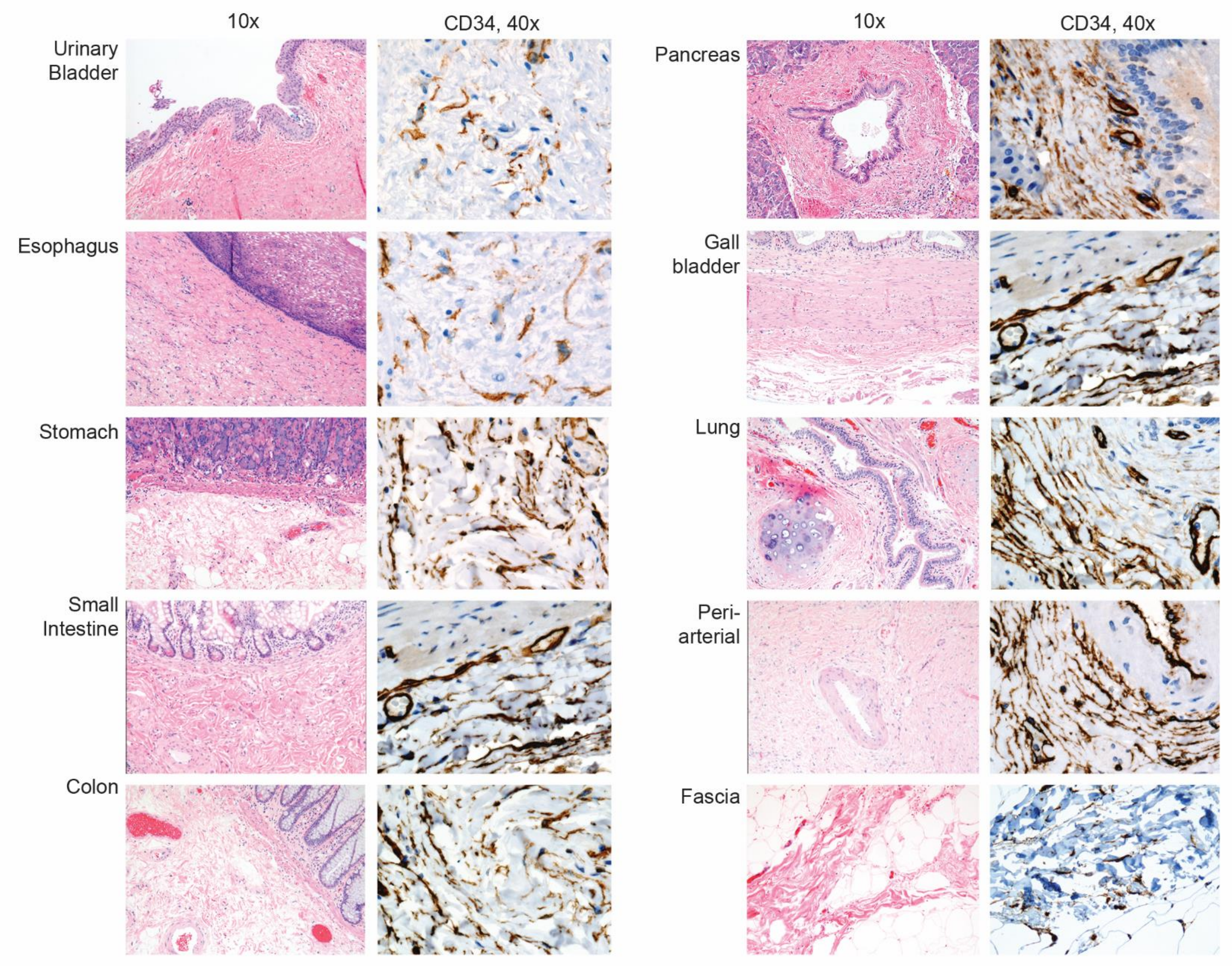

Supplemental Figure 1. Interstitial spaces and collagen bundle lattices in diffuse fibroconnective tissues of the body.

All H&E images are magnification 10x and show pink connective tissue in which collagen bundles are collapsed, though artifactually narrowed interstitial spaces are still recognizable in all tissues. Immunostaining for CD34 in all these tissues (40x) highlights the positive lining cells (brown, DAB) that lie on the glassy, pale blue (hematoxylin counterstain), acellular collagen bundles. Often the cells seem separated from each other and opposite surfaces of the collagen bundles often show no adherent cells at all. Submucosae of the urinary bladder, tubular digestive tract (esophagus, stomach, small intestine, colon), pancreatic ducts, and gallbladder, bronchial tissues of the lung

(bronchial submucosa and pericartilagenous tissue), peri-arterial connective tissue and fascia are illustrated. Portal tract stroma (peri-vascular and peri-biliary) of the liver and peri-venular connective tissue show the same features (not shown).

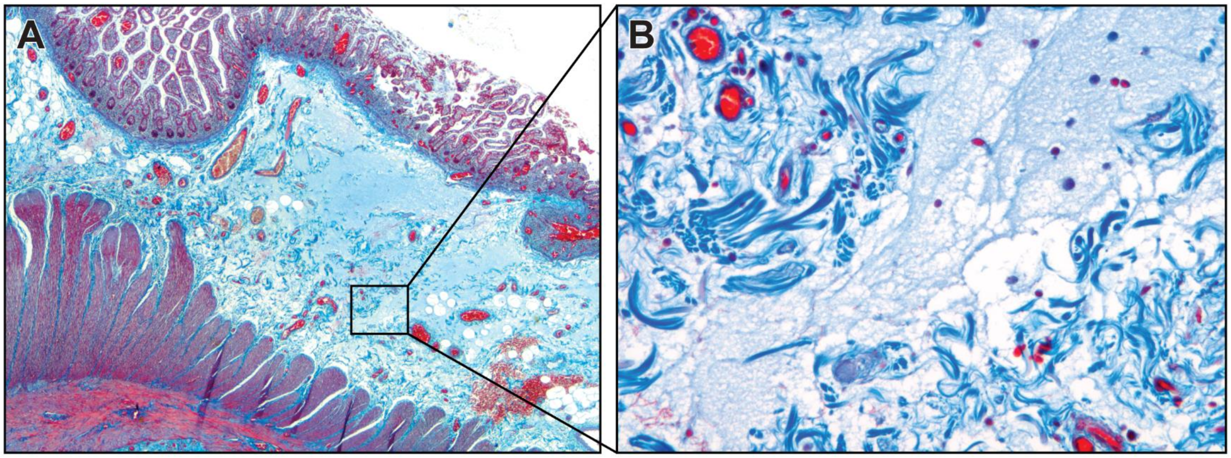

Supplemental Figure 2. Distention of the interstitial space in response to small bowel obstruction in specimens of small intestine resected for incarceration in a ventral hernia (representative image, n=6 patient samples). **(A)** Submucosa proximal to the obstruction shows dilatation of the interstitium and splaying and spreading of collagen bundles. The space between them suggests distension with increased interstitial fluid proximal to the obstruction (chromotrope aniline blue, 2x). **(B)** Higher power view of boxed region in **A** shows the pale blue-grey, granular and bubbly proteinaceous fluid between spread dark blue collagen bundles (chromotrope aniline blue, 10x).
